# Fatal Late-Onset Ornithine Transcarbamylase Deficiency in an Adolescent: A Case Report

**DOI:** 10.7759/cureus.107721

**Published:** 2026-04-26

**Authors:** Evangelos Christou, Katerina Ntouzepi, Maria Machaira, Konstantinos Tziouvas, Ari Patsoura

**Affiliations:** 1 Pediatric Intensive Care Unit, General Children's Hospital Panagiotis and Aglaia Kyriakou, Athens, GRC

**Keywords:** adolescent, coma, hyperammonemia, late-onset otc deficiency, rare cause of altered mental status

## Abstract

The late-onset phenotype represents the most prevalent form of ornithine transcarbamylase (OTC) deficiency and is characterized by marked clinical heterogeneity, with disease onset ranging from early infancy to adulthood. We describe the case of a 12-year-old female with no significant past medical history who presented to the Emergency Department with an impaired level of consciousness and profound hyperammonemia (plasma ammonia: 432 μg/dL, and impairment of consciousness). Despite early initiation of continuous renal replacement therapy and aggressive management of cerebral edema, the patient developed refractory neurological deterioration and died five days after presentation. This case highlights that the late-onset form of OTC deficiency may manifest with severe neurological involvement and may be life-threatening. Timely recognition and aggressive management of hyperammonemia and impending hyperammonemic coma are essential to achieve improved clinical outcomes.

## Introduction

Ornithine transcarbamylase (OTC) deficiency constitutes a member of the group of rare inborn errors of metabolism collectively designated as urea cycle disorders (UCD), which currently comprise seven additional distinct entities [1]. Defective ammonia detoxification with resultant systemic and cerebral ammonia accumulation induces astrocyte swelling, thereby precipitating the development of cerebral edema [2].

All UCD follow an autosomal recessive pattern, with the exception of OTC deficiency, which exhibits an X-linked recessive inheritance. The estimated prevalence of the disease ranges from 1:14,000 to 1:80,000 [3]. Owing to its X-linked recessive inheritance, the disorder predominantly manifests as a severe, early-onset phenotype in hemizygous males. In heterozygous females, X-chromosome inactivation, including skewed patterns, may result in residual gene activity and partial enzymatic deficiency. Nevertheless, due to significant phenotypic variability, approximately 10-20% of female carriers who are otherwise considered asymptomatic may develop clinical manifestations at any age [3].

Late-onset OTC deficiency shows marked clinical heterogeneity, with symptom onset possible at any age; hemizygous males and about 20% of heterozygous females remain at risk for hyperammonemia-induced manifestations. Life-threatening hyperammonemic crises are frequently precipitated by catabolic stressors, including intercurrent infections, fever, prolonged fasting, surgery, trauma, pregnancy, or the postpartum period, which increase endogenous protein breakdown and nitrogen load, thereby overwhelming residual urea cycle capacity and leading to acute metabolic decompensation [4]. Ammonia accumulation exerts direct neurotoxic effects, primarily through astrocyte glutamine accumulation, and alterations in cerebral energy metabolism. The resulting clinical spectrum commonly includes recurrent vomiting consistent with cyclic vomiting syndrome, hyperammonemic encephalopathy, migraine-like headaches, and a wide range of neuropsychiatric disturbances [5,6].

We report the case of a 12-year-old female with late-onset OTC deficiency who required admission to the Pediatric Intensive Care Unit (PICU) due to hyperammonemic encephalopathy. Although rare, OTC deficiency should be considered in young females with unexplained coma and hyperammonemia. Prompt diagnosis and initiation of metabolic therapy are crucial to prevent irreversible neurological damage and improve outcomes.

## Case presentation

History

A 12-year-old female with no significant past medical history was admitted with an impaired level of consciousness. Her family history was notable for a mother diagnosed with bipolar disorder under ongoing pharmacotherapy. The patient’s symptoms commenced 24 hours prior to admission, with a progressive decline in level of consciousness accompanied by recurrent episodes of vomiting. Based on the available clinical information, no identifiable precipitating trigger leading to the manifestation of OTC deficiency was identified in this patient. On arrival at the Emergency Department, neurological assessment revealed a depressed level of consciousness, with a Glasgow Coma Scale (GCS) score of 9/15. Empirical administration of flumazenil was undertaken as a reversal agent for suspected benzodiazepine exposure, but no clinical improvement was observed. Written informed consent for publication was obtained from the patient’s mother.

Clinical course

In view of her worsening neurological status, the patient was emergently transferred to the PICU for advanced monitoring and supportive management. Upon admission, she was normothermic and hemodynamically stable, albeit hypertensive and tachycardic, with a blood pressure of 165/82 mmHg and a heart rate of 139 beats per minute. Peripheral oxygen saturation was 97% while receiving supplemental oxygen at 2 L/min via a face mask. Initial laboratory evaluation, including complete blood count, comprehensive metabolic panel, and coagulation profile, was unremarkable, with the notable exception of markedly elevated plasma ammonia levels (NH₃: 432 μg/dL, normal range 10-90 μg/dL). Arterial blood gas analysis demonstrated mild respiratory alkalosis. The patient was intubated within 24 hours due to progressive neurological decline. Her hemodynamic status remained stable. Cranial CT demonstrated early cerebral edema. Immediate interventions to mitigate cerebral edema included continuous intravenous administration of 3% hypertonic saline and elevation of the head to 30°. The patient experienced episodes of generalized tonic-clonic seizures, which were managed with intravenous midazolam, and was initiated on continuous 24-hour electroencephalographic monitoring. Urine and blood toxicology screenings for narcotic and psychoactive substances were negative.

**Figure 1:**
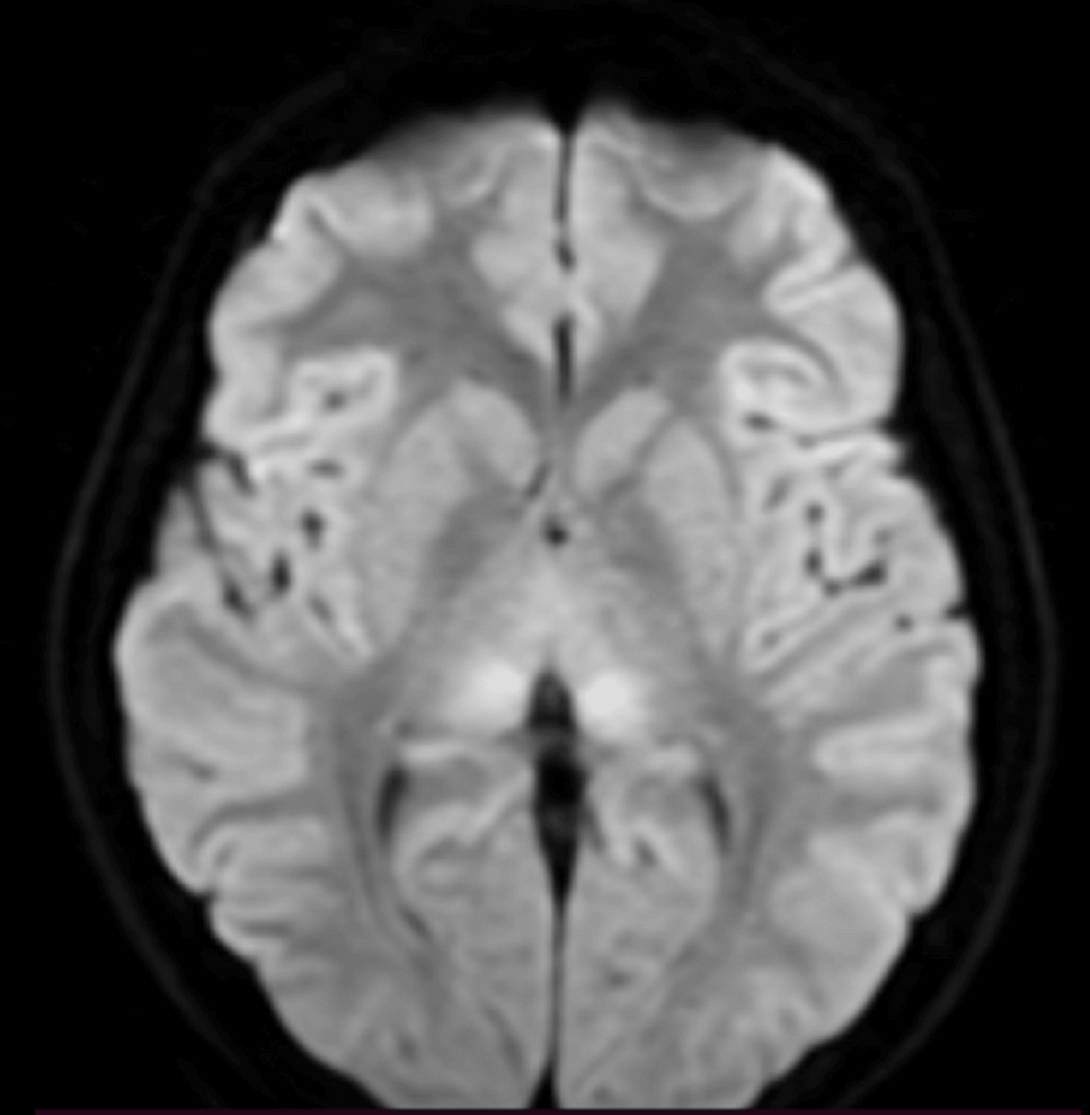
MRI brain revealed areas of hyperintense signal with symmetric involvement of the posterior thalami.

**Figure 2:**
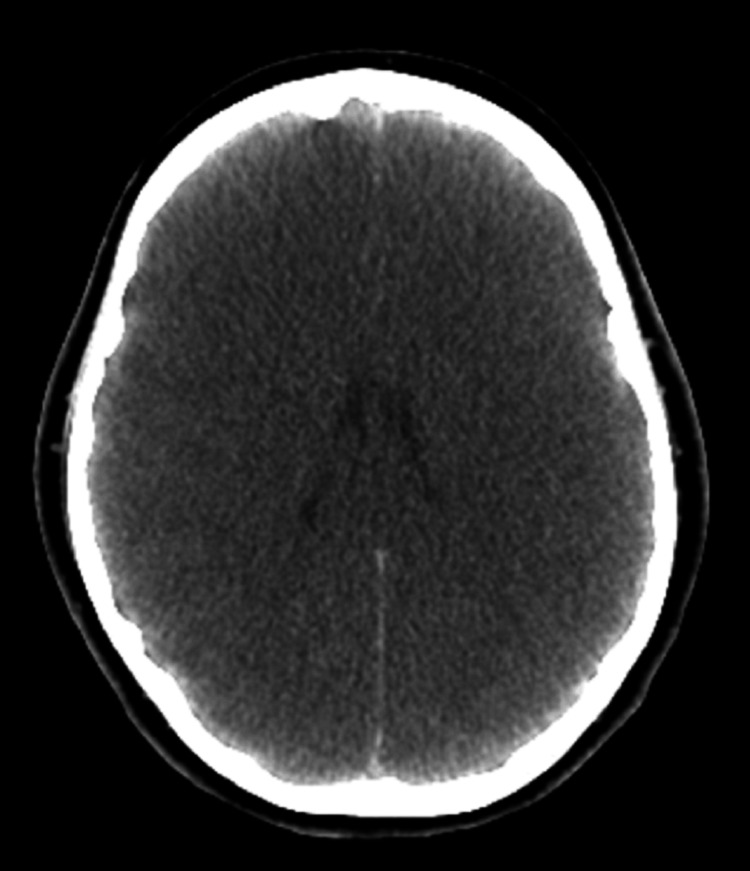
CT brain revealed evidence of cerebral herniation.

Metabolic investigation, including plasma amino acid analysis and urinary organic acid profiling, demonstrated markedly reduced plasma citrulline levels, elevated glutamine concentrations, and markedly increased urinary orotic acid excretion. In the context of severe hyperammonemia, continuous intravenous administration of sodium benzoate and sodium phenylbutyrate was initiated within the first hours after admission. Owing to an insufficient clinical response, continuous renal replacement therapy (CRRT) was initiated on hospital day 2. Furthermore, the MRI brain revealed areas of hyperintense signal with symmetric involvement of the posterior thalami, accompanied by imaging features indicative of diffuse cerebral edema (Figure 1). An intracranial pressure (ICP) monitoring catheter was placed. On hospital day 4, despite normalized ammonia levels and ongoing CRRT, the patient developed elevated ICP, hypertension, and bradycardia. Repeat CT brain revealed evidence of cerebral herniation (Figure 2). Brain death testing was subsequently performed, and the patient succumbed on the fifth 24-hour. Whole exome sequencing revealed that the adolescent harbored a pathogenic variant in the OTC gene. She carries a heterozygous mutation in exon 8 at position c.830>A, resulting in the amino acid substitution p.(Arg277Gln).

## Discussion

Systemic ammonia is generated predominantly in the intestinal mucosa through glutamine deamination by enterocytes and bacterial urease activity, with additional contributions arising from skeletal muscle metabolism and increased protein catabolism [7]. Hepatic detoxification constitutes the principal pathway for ammonia clearance. Within periportal hepatocytes, ammonia is incorporated into carbamoyl phosphate by the mitochondrial enzyme carbamoyl phosphate synthetase I, in an N-acetylglutamate-dependent manner, thereby initiating the urea cycle. Through sequential enzymatic reactions, nitrogen is ultimately converted to urea, which is subsequently eliminated via renal excretion [8].

OTC plays a pivotal role in the urea cycle by catalyzing the mitochondrial condensation of carbamoyl phosphate with ornithine to form citrulline, a critical intermediate in nitrogen disposal. OTC is a mitochondrial matrix enzyme predominantly expressed in hepatocytes and, to a lesser extent, in enterocytes of the small intestine; however, in humans, functionally significant activity is confined to the liver, where it plays an essential role in ureagenesis [9].Τhe OTC gene is located on the short arm of the X chromosome (Xp21.1) and encodes a 354-amino acid precursor protein with an approximate molecular weight of 40 kDa, which undergoes mitochondrial import and proteolytic processing to yield the mature enzymatically active form [10]. OTC deficiency exhibits a broad phenotypic spectrum, from severe neonatalonset hyperammonemia with high mortality to late-onset forms manifesting at any age. Heterozygous females may remain asymptomatic due to residual enzyme activity [11]. In this case, the patient had an unremarkable past medical history prior to disease manifestation. Notably, her mother has a history of bipolar disorder; however, carrier status for OTC deficiency has not been genetically confirmed. This suggests possible unrecognized heterozygosity, considering the phenotypic variability and potential neuropsychiatric manifestations of partial enzyme deficiency. Although disease manifestation in previously asymptomatic individuals has been associated with precipitating factors such as hypercatabolic states, increased nitrogen load, heightened metabolic demand on the urea cycle, and physiological stressors, no identifiable triggering event was documented in the present case [12,13].

Hyperammonemia represents a potentially reversible cause of cerebral edema and encephalopathy and should be systematically considered in the differential diagnosis of patients presenting with unexplained alterations in level of consciousness. Ammonia readily crosses the blood-brain barrier and is subsequently metabolized within astrocytes to glutamine via glutamine synthetase. Excessive intracellular accumulation of glutamine increases osmotic load, leading to astrocytic swelling secondary to osmotic water influx and, ultimately, to elevated ICP [14]. These mechanisms underscore the necessity for immediate therapeutic intervention aimed at rapid ammonia reduction, as plasma concentrations exceeding 1000 μg/dL have been associated with adverse neurological outcomes and increased mortality [15]. In this case, initial treatment consisted of intravenous nitrogenscavenging therapy with sodium benzoate and sodium phenylbutyrate; however, due to insufficient biochemical response, CRRT was instituted. Although plasma ammonia levels subsequently normalized, prolonged periods of marked hyperammonemia were strongly associated with the development of severe and irreversible encephalopathy in our patient. In instances of rapidly escalating plasma ammonia or concentrations exceeding 400 μg/dL, in the absence of definitive management guidelines, initiation of CRRT is frequently employed to achieve prompt biochemical normalization and to prevent the development of ammonia-induced neurotoxicity [16]. 

## Conclusions

This case underscores the necessity of maintaining a high index of suspicion for OTC deficiency in previously asymptomatic individuals presenting with unexplained alterations in consciousness accompanied by elevated plasma ammonia levels. Despite timely and appropriate management, the outcome was poor, underscoring the severe neurotoxic and systemic impact of hyperammonemia.
